# Micromachining of Invar Foils with GHz, MHz and kHz Femtosecond Burst Modes

**DOI:** 10.3390/mi11080733

**Published:** 2020-07-29

**Authors:** Simas Butkus, Vytautas Jukna, Domas Paipulas, Martynas Barkauskas, Valdas Sirutkaitis

**Affiliations:** Laser Research Center, Faculty of Physics, Vilnius University, Saulėtekio Ave. 10, LT-10223 Vilnius, Lithuania; vytautas.jukna@ff.vu.lt (V.J.); domas.paipulas@ff.vu.lt (D.P.); martynas.barkauskas@ff.vu.lt (M.B.); valdas.sirutkaitis@ff.vu.lt (V.S.)

**Keywords:** femtosecond micromachining, burst processing, GHz, MHz, micromachining, Invar

## Abstract

In this work, a burst mode laser is used for micromachining of 20 µm–250 µm thick Invar (Fe64/Ni36) foils. Holes were drilled by firing multiple pulses transversely onto the sample without moving the beam (percussion drilling). The utilized laser system generates a burst of a controllable number of pulses (at 1030 nm) with tunable pulse-to-pulse time spacing ranging from 200 ps to 16 ns. The sub-pulses within the burst have equal amplitudes and a constant duration of 300 fs that do not change regardless of the spacing in time between them. In such a way, the laser generates GHz to MHz repetition rate pulse bursts with a burst repetition rate ranging from 100 kHz to a single shot. Drilling of the material is compared with the non-burst mode of kHz repetition rate. In addition, we analyze the drilling speed and the resulting dependence of the quality of the holes on the number of pulses per burst as well as the average laser power to find the optimal micromachining parameters for percussion drilling. We demonstrate that the micromachining throughput can be of an order of magnitude higher when using the burst mode as compared to the best results of the conventional kHz case; however, excess thermal damage was also evident in some cases.

## 1. Introduction

Due to the high precision and low thermal damage offered by ultrashort pulse micromachining, femtosecond laser systems are widely applied in both industrial and scientific fields [1]. Though the advantages of such systems (if compared to longer pulse or non-laser-based micromachining [2]) are unquestioned, the constantly advancing industry requires higher micromachining throughput, better end-quality and higher versatility in terms of machinable materials [3,4,5]. To accompany the needs of the industry, a trend towards high average power (>100 W) femtosecond laser systems development and utilization for micromachining has become apparent [6,7]. In general, higher pulse average power leads to an increase in processing throughput, but maintaining the efficiency and quality of the process comparable to lower power systems is posing a significant challenge [8]. High average power lasers produce pulses of higher energy that increase the fluence striking the material, reaching values exceeding the ablation threshold by more than two orders of magnitude. Such a situation is not preferred for precise micromachining because the absorbed energy is transferred to the kinetic energy of the ablation debris or/and the shock wave resulting in the reduction of process quality and a drop in material removal efficiency [9]. A possible workaround could be the use of spatial light modulators (SLM) [10,11,12] or various diffractive optical elements (DOE) that enable fluence redistribution and parallel processing. However, this approach is not always applicable as these methods are application-specific, increase overall laser system complexity, and could have low damage threshold or efficiency. A different approach could be used if the high average power laser system operates at high repetitions rates (>10 MHz). In this situation, the fluence may be set near the optimal ablation setting as described in [13]; however, the pulse-to-pulse time separation becomes small enough for heat accumulation to significantly decrease the end-quality of the micromachined samples [14,15]. It is especially visible at the Gaussian beam periphery and is a typical problem for high average power systems. This outcome stems from the fundamental properties of light-matter interaction and cannot be easily overcome. Though the quality of the micromachined samples may be improved by increasing the spatial separation (decreasing pulse overlap), this requires using high-speed (10–100 m/s) translation systems, which are either specially modified galvo-scanner setups or polygon scanners [16]. This setup presents several drawbacks: implementation of sharp focusing in the system becomes complicated, and loss of working area cannot be avoided. For the case of the polygon scanners, vector scanning is near impossible, leaving these systems applicable only for specific pattern machining.

Recently, a new technique, burst mode processing, was demonstrated for micromachining materials with far better efficiency than the conventional systems [17,18,19,20,21,22]. The stated technique utilizes the separation of energy of a single pulse into multiple pulses (burst) by a certain time delay. The separation time can vary from ~100 ps to ~10 ns. In this case, the relatively high pulse energy can be divided into a packet of pulses with pulse energy values that are closer to the optimal ablation rate setting. In addition, a train of pulses can alter the properties of the material as the sequence of pulses impinges on the sample, i.e., if the temporal separation is short enough that the heat does not diffuse away until the second pulse within the burst arrives, the heat can start to build up locally in the impinged region. In this case, given the right conditions, the reflectivity of the metal can decrease from >95% (reflection at room temperature for the first pulse) to <80% (for the trailing pulse) if two pulses are separated by ~100 ps [23]. The penetration depth of light is also altered, which may reduce thermal damage and ablate more material per unit of energy [24], especially when considering deep craters. Therefore, from purely a theoretical point of view, the ablation of materials with the burst of femtosecond pulses should be a more efficient process; hence, the amount of ablated material per unit of time per unit of energy should increase. However, modeling of the absorption of energy and pulse propagation in time and space is a complicated task since one should take into account the presence of the plasma after the first pulse. Numerous reports [25,26,27,28] suggest that after a femtosecond pulse hits the surface of the sample, the ablated mass, which in that point in time is a mixture of hot electrons, ions and nanoparticles, disappears from the vicinity of the target region in a time scale of <10–100 ns. Therefore, it is not clear whether related plasma effects such as plasma reignition (absorption of impinging photons by the previous-pulse generated plasma which has not yet fully relaxed), beam scattering, defocusing and prolonged plasma-target coupling may have some influence on the micromachining process in terms of throughput and end-quality [29,30,31,32]. 

In this work, we present a study on the micromachining throughput of Invar sheets having different thicknesses (20, 50, 150, 250 μm) using a femtosecond laser that has an innovative multi-burst regime. This femtosecond laser operates in different burst modes, i.e., it can supply a controlled train of pulses every 10 μs (100 kHz carrier frequency). The number of pulses within each burst can be varied (up to 12 pulses/burst), and the time delay between the pulses can also take on different values; therefore, a situation can be realized where the pulses are spaced at a time scale that translates to GHz (200 ps time delay), MHz (16 ns time delay) and kHz (10 µs) repetition rates while keeping the laser at a constant average power. In this work, we perform an experimental investigation on percussion drilling of through-holes where we vary the average laser power, number of pulses/bursts, and the time delay between consecutive pulses within the burst.

## 2. Materials and Methods

**Figure 1:**
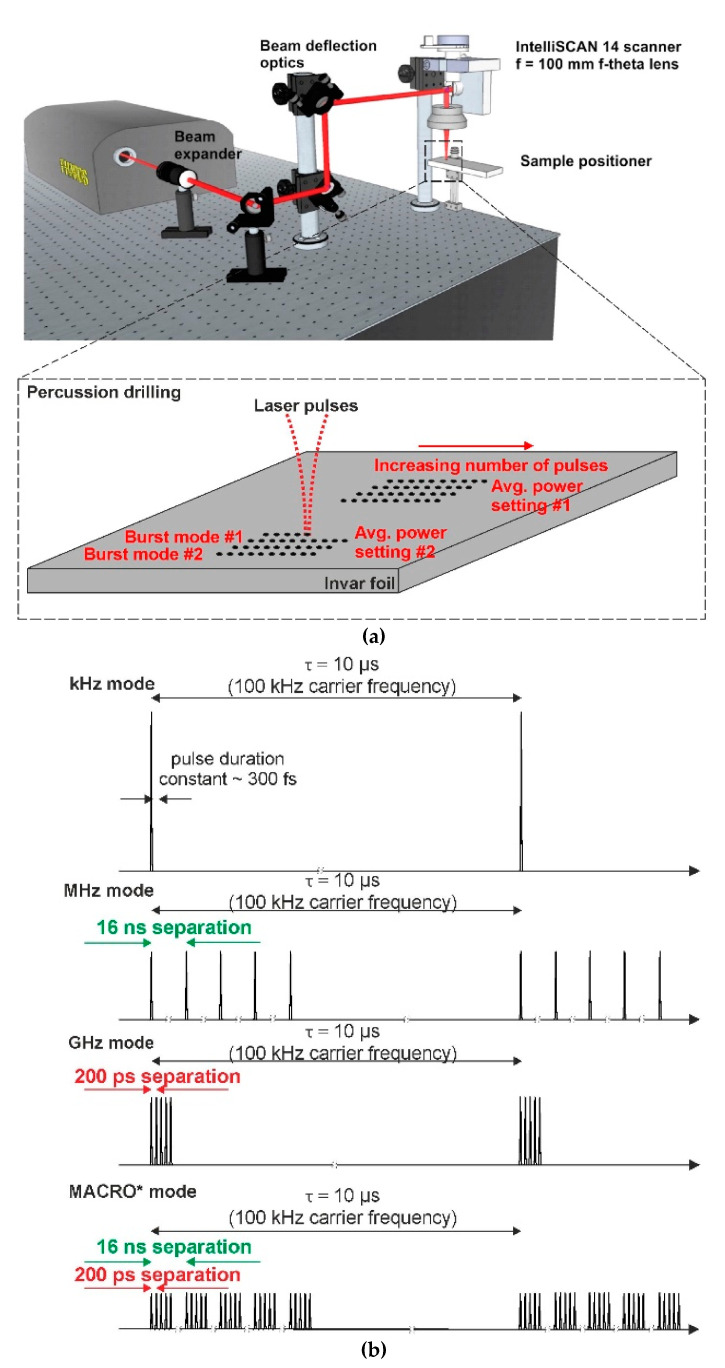
(**a**) experimental setup for Invar foil percussion drilling. The bottom part shows the fabrication algorithm used. (**b**) illustration of the burst modes generated by the laser. The 16 ns temporal separation equates to a MHz, whereas the 200 ps to a GHz repetition rate. The MACRO mode is a combination of the MHz and GHz modes and produces a number of pulses temporally separated by 200 ps, which are grouped by identical trains spaced by 16 ns. The integrated energy per cycle is the same for all the different burst modes.

A multi-burst laser Pharos^®^ (Light Conversion Ltd., Vilnius, Lithuania) was used to produce bursts of femtosecond pulses temporally separated in the ~100 ps–10 ns time scales with a central wavelength of 1030 nm. The maximum average power of the laser was 5 W. The burst of femtosecond pulses could be generated at a repetition rate ranging from 100 kHz to a single shot, and a burst of pulses can be produced with separation times ranging from 200 ps to 15 ns. In addition, a combined packet of pulses separated by both 200 ps and 15 ns can also be realized, and this was called the MACRO mode. The illustration of the bursts and its modes are depicted in Figure 1. The maximum number of pulses within the burst was limited by the hardware and was equal to 12 pulses. The energy of each pulse within the burst in the whole average power range was measured using the time-correlated single-photon counting method [33] and had a variation of no more than 10% standard deviation. Using a galvo-scanner (IntelliScan14, Scanlab Ltd., Puchheim, Germany) and an f = 100 mm F-theta lens, the pulses were focused on the sample. The experimental setup is shown in Figure 1a. The beam waist was positioned directly on the surface of the sample, and the position was fine-tuned by firing single pulses on the sample at different heights (at a step of 0.2 mm) until the smallest diameter crater was achieved. The spot size at the focal position was measured with a CCD camera and was equal to 22 µm (FWHM). The Rayleigh length for this beam was calculated to be ~1.1 mm. Since the Rayleigh range is significantly longer than the thickness of the investigated thickest sample, changing the focal position while processing was not needed. The polarization of the beam was converted to circular using a λ/4 wave-plate, which was placed in front of the scanner. The investigated material was Invar (64Fe36Ni) foils having thicknesses of 20 µm, 50 µm, 125 µm and 250 µm. The dimensions of each sheet were 10 cm × 10 cm. Two sides of the foil sheet were fixed to clamps, which were connected with a tensioning rod, and by increasing tension between the clamps, a flat foil surface was produced (the surface roughness of the foil was measured using an optical profiler and was 70 nm (arithmetic mean deviation–Ra)). The used samples were homogenous and free of crests. To account for sample warping, we used a smaller surface area at the center to carry out the micromachining experiments (5 cm × 5 cm), since the material near the clamps would be considerably stiffer. The sample holder was leveled perpendicular to the F-theta lens. The number of pulses required to drill through the material with the different burst mode settings was recorded while varying the average laser power (total energy emitted per second). It is worth mentioning that for each burst mode setting, the energies of the individual pulses are different given a specific average power. The pulse energy is important as it determines the fluence and the maximum depth at which the material can be drilled. In this study, we did not perform drilling tests using constant energy values as it would require a change of average power and the sample irradiance would not be constant at each processing mode. Taking into consideration that Invar is known as a poor thermal conductor (approximately 10 Wm^−1^K^−1^ or two orders of magnitude lower than aluminum), the outcomes due to the heat effect could produce unclear results. Therefore, comparing the results acquired at the same average power levels allows the micromachining throughput to be assessed and the least heat effected scenario to be determined. The ablated volume was measured using an optical profiler (PLµ 2300, Sensofar, Barcelona, Spain). 

The experiment was carried out in the following manner: the fabrication algorithm consisted of producing an array of spots on the sample, each separated by 300 µm (which is roughly an order of magnitude larger than the laser affected area of the surface) and corresponding to a different number of pulses in an increasing order, as shown in the bottom part of Figure 1a. The step at which the number of pulses was increased per each location was different for different parameter sets. As will be shown shortly, it may take only several pulses/bursts to produce through-holes with a particular set of parameters at an average power setting of 5 W. However, it may take hundreds of pulses/bursts to create a through-hole with a different set. Therefore, the step was adjusted as 1/10 of the final number of pulses required to produce a through-hole, i.e., if 10 pulses/bursts are needed to make a through-hole, the step is 1 pulse, and if 1000 pulses/bursts are needed, then the step is 100 per each location. In total, hundreds of holes were produced to gather the necessary data. When the arrays of craters have been created, the samples are removed from the processing station and firstly inspected with an optical microscope. The image from a microscope used in transmission mode clearly shows the data-points that had through-holes. The process is repeated several times, and the number of required pulses to drill through the material is retrieved. The analysis of the ablated volume dependence on laser energy was done for several chosen energy parameters of six burst modes and one reference mode. The points of interest are inspected with an optical profiler in topographic mode, i.e., the ablated volume is measured by slicing through the planes in the Z direction. It is worth noting that partial hole formation (not fully drilled holes) was not investigated due to the measurement equipment limitations. The different laser operation modes were compared with the standard 100 kHz (single pulses separated in time by 10 µs) processing case, which acted as reference mode. Note that we limited our analysis to the case when the carrier frequency (inverse of time between two bursts) remained constant at 100 kHz throughout the experiments regardless of the burst setting (ns separation or ps separation). Six different burst mode parameters were chosen for the investigation: 5, 10 and 12 pulse bursts with separation of 200 ps, 5 and 8 pulse bursts with separation of 16 ns and MACRO mode (3 burst pulses separated by 16 ns containing 11 pulses separated by 200 ps). Lastly, the traditional single pulse processing at a 100 kHz repetition rate (10 µs pulse separation) was tested for comparison purposes (see Figure 1b).

## 3. Results

**Figure 2:**
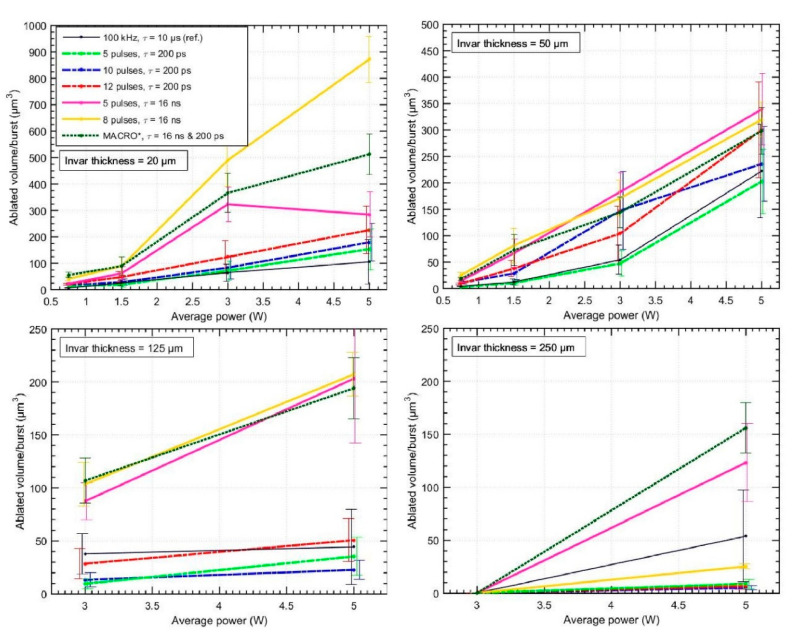
Ablated volume per burst vs. different average laser power. The tests were conducted on 20–250 µm thick Invar sheets varying the different burst modes. The different colors represent different burst modes. The removed volume is measured only for a through-hole that has a ratio between the hole entrance and exit diameter of at least two. Note the change in *y*-axis scale in the results.

The foils were percussion drilled and the number of pulses required to drill through the material as well as the dimensions of the ablated holes were investigated. The removed volume was recorded only for the cases when a through-hole was formed in the foil. When the through-hole is produced, the dimensions of the hole at the bottom of the sample may only be several µm and increases as the number of impinging pulses increases. Due to the typical industrial need to produce holes with an entrance-exit ratio which is lower than two, we have only considered through-holes that meet this criterion, expelling highly cone-shaped holes that potentially exhibit irregularities in the shape of the crater or the exit hole shape. This estimation limits the minimum number of pulses needed to produce through-holes and many holes were disregarded as not meeting this criterion when the hole was just starting to appear. The ablated volume per pulse (or single burst) dependence on the average laser power is shown in Figure 2. The error bars depict two standard deviations from the mean value and were calculated by conducting experiment replicates (five replicates per each experimental point were performed). Figure 2 shows that the error bars are longer for the higher average power settings. This is attributed to the slight melting of the material and crater formation on the surface, which leads to deviation from the ideal hole shape and less replicable at different positions of the sample. Therefore, the difference in shape also leads to a larger variation in ablated volume. This is also more pronounced for the thinner materials as the heat dissipates slower in thin samples since heat conduction in the metal-air interface is lower as compared to the metal itself. Additionally, the small number of pulses that are required to drill through the thin (20 µm) sample as compared to the thick (250 µm) sample may play a role as well in shape deviations. It is worth pointing out that even though ablation produced with femtosecond pulses is generally considered as a non-thermal process, a considerable amount of heat is transferred into the material during percussion drilling of deep craters with high repetition rate lasers. The laser fluence on the crater walls drops due to the increased surface area of the crater and can become lower than the ablation threshold; therefore, absorbed light energy is transferred into thermal energy, which heats the material around the crater.

The results indicate that higher ablated volume per burst values are achieved for thinner samples. This is attributed to the smaller decrease in fluence at the surface of the material when a crater is produced, and this has a direct impact on the reduction of the material removal efficiency. The walls of the deep crater have a larger surface area on which the beam is projected, which results in fluence drop. If comparing the total surface area of the hole sidewalls drilled in the case of the 20 µm and 250 µm samples, the difference in surface area is >10-fold. Therefore, for deeper holes, the drop in fluence is much more pronounced. The detailed dynamics of the crater formation were not studied here; however, it is known that as the successive pulses impinge the same surface area, the surface gradually changes from a flat to a V shape. When more and more pulses hit the surface, the fluence decreases from its initial value and may go down below the ablation threshold. In that case, the overall ablation efficiency is low. Additionally, as the channel becomes deeper, the light incidence angle on the crater walls changes, which also affects the absorption properties. In the forthcoming discussion, the V-groove formation dynamics are regarded as similar for all of the different burst modes, and only the increase in surface area is considered to be the main limiting factor that contributes to ablation efficiency decrease. More information regarding the intricacies of V-shape formation may be found in [34]. 

It was noticed that burst modes with the 16 ns separation (MHz regime, plotted as solid lines) remove the most volume per burst. Only 8 pulses at the 5 W setting are required to drill through the 20 µm sample; this translates to a rapid fabrication rate equal to 1 hole/80 µs. For thin samples (20, 50 and 125 µm), the 8 pulses/burst separated by 16 ns setting yields the faster fabrication throughput, though, for the thicker sample (250 µm), the 5 pulses per burst setting is superior. We attribute this result to the same V-shape groove formation effect that forms during multiple pulse impingements. Since the integral energy/burst is equal for all the cases at a specific average power setting, the higher number of pulses within the burst translate to a lower fluence setting per pulse. If the surface area of the V shape becomes large enough (the crater becomes deep), the fluence decreases from its initial value to the point where it is below the ablation threshold, as described previously. For the 5 pulses/burst setting, this condition is reached at a larger depth; therefore, fewer pulses per burst are preferred for thicker samples provided that the average power is the same. It was noticed that no through-holes were produced when the ablating sheets were thicker than 125 µm, with laser power values below 3 W with the 8 pulses/burst and the 12 pulses/burst setting. These points were discarded from the investigation because they are not directly comparable to the other modes. However, a trend is visible, which is that ablation per unit of energy is more efficient when a higher number of pulses are used in the burst, though they are not always usable due to practical reasons (thick materials need to be processed).

The results also demonstrate that in almost all the cases, the burst of pulses with the MHz repetition rate (16 ns sub-pulse separation) is superior when considering the removed material volume per unit of burst energy. Additionally, the burst in the GHz case (200 ps sub-pulse separation pulses, plotted as point-dash lines in Figure 2) is more efficient than the traditional kHz case (solid black line in Figure 2) for thin samples up to a thickness of 50 µm, while for the thicker samples the efficiency is similar or even lower than the kHz case. There may be multiple reasons leading to this outcome. In theory, a comparison of solely absorption and reflection of energy within the volume should lead to a result where the GHz case is more efficient due to the higher absorption and/or lower reflection (order of ~10%) of hotter metallic surfaces. Since the pulse-to-pulse time is short (200 ps), the trailing pulses within the burst impinge onto a hotter surface as compared to the MHz case. This is due to the fact that the hot area around the central peak does not have sufficient time to diffuse further into the material until the next pulse arrives, as described by Ilday et al. [18]. Subsequently, this should lead to larger ablated volumes (as is predicted for the case of copper [23,35]) and a lower thermal damage threshold. However, we speculate that this situation may not always be achievable and could be material-dependent (different for melting, non-melting materials). Additionally, regarding the heat dissipation, there is a need to take into account a portion of the material that is removed in the form of hot atoms, ions, electrons and nanoparticles, after each pulse in the burst hitting the surface. The relative percentages of these species may change depending on the processing conditions (energy, carrier frequency, focusing conditions). As stated in the introduction section, the plasma cloud stays in the vicinity of the ablated crater far longer than 200 ps; therefore, the second pulse (within the burst) interacts partly with the plasma cloud as well as the ablated crater surface. A portion of energy may be absorbed or defocused by the plasma cloud (caused by variation of the complex refractive index due to the variation of plasma density). In addition, under some irradiation conditions, the plasma may reach a reflective state, in which case a part of the energy may be reflected from the sample [36], while the remaining energy will generate an additional plasma cloud, which is launched from the surface. Since the number of pulses is high in the investigated cases, it is a challenge to predict the dynamics of the plasma and plasma-pulse-matter interaction on an interburst scale, and more fundamental research is required to answer this question. The experimental results show that the plasma screening outweighs the advantages of processing a hotter surface with subsequent pulses in the burst and removes less volume per unit of energy (and per unit of time, since the carrier frequency is the same for all of the investigated cases) for the GHz case. It is contemplated that pulses within the burst in the MHz case are not efficiently screened by the plasma, and the surface at the target area is still relatively hot, enabling slightly faster material removal rates.

The material ablation results with the so-called MACRO pulse mode (3 sub-bursts separated by 16 ns comprised of 11 pulses separated by 200 ps, plotted as a dot-line) appears somewhat unusual. In total, the MACRO mode is comprised of 33 pulses separated in time by different time scales. The fluence per single pulse within the bursts has the smallest value in comparison to the other cases; therefore, this mode should be largely affected by the V shape that forms during multiple pulse impingement. However, our results show that the contrary is true and hints to the possibility that plasma reignition and, possibly, the previously mentioned plasma-target coupling play an important role when ablating with the MACRO mode. Such an ablation regime may be regarded as similar to nanosecond processing. From the results provided in Figure 2, the MACRO mode shows most versatility in being close to the best performance in all the thicknesses of the samples and is best suited for thick (>200 µm) material micromachining.

It is worth mentioning that all of the burst modes (MHz, GHz and MACRO mode) are more efficient in removing volume per unit of energy as compared to the conventional 100 kHz setting in the investigated case. Though as stated, if the material is thick enough, through-hole fabrication may become impossible due to the lower fluence per pulse that is within the burst when a V-shape crater forms. In addition, the higher the number of pulses within the burst, the higher the average power that is required to efficiently micromachine the material. When varying the average power, there will always be a setting when the removed volume/unit of energy and unit of time is higher for the burst modes. Because the samples were percussion drilled, the crater becomes deeper after each pulse. Since the surface area increases (fluence decreases) for each subsequent pulse, we do not observe an optimal average power or fluence setting for any of the cases that are out of the error range. It is worth mentioning than the ablation of the material and formation of the crater starts immediately after a single pulse or burst when drilling the Invar foils. The damage threshold values for the different burst modes were not investigated in this article, since under the studied irradiation conditions, the beam fluence was higher than the damage threshold; however, it is expected that the damage threshold when micromachining with burst will be lower, as compared to the single-pulse case, due to thermal accumulation effects. This suggests that the lower reflection values and higher absorption initiated by the burst modes increases the fabrication throughput, even if the negative effects of the plasma-beam interaction are present. Since the MHz regime appears more efficient than the GHz regime, perhaps by reducing the unwanted plasma effects, the processing rate may further be improved. Therefore, it is believed that a different spacing of the pulses (somewhere in between the investigated 200 ps and 16 ns range) may be better for hole drilling. Such conditions would still sustain the higher absorption/lower reflection scenario, while the interaction of the beam with the plasma would be minimized.

**Figure 3:**
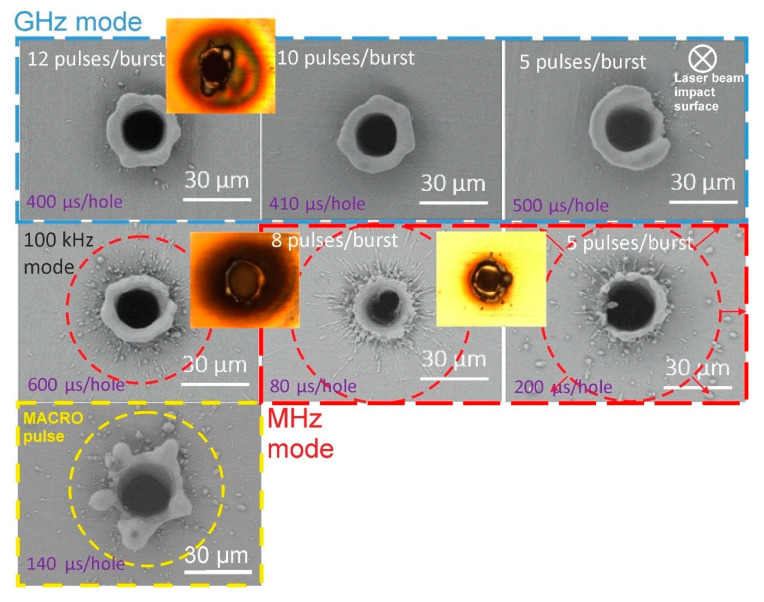
SEM images of the material surface when through-holes are produced in a 20 µm thick sample for different burst pulse modes, which are indicated by different color borders namely blue for GHz, red for MHz, yellow for MACRO, and for comparison, the 100 KHz case is presented without the border. The laser power was set to 5 W in all the cases and the time needed to produce these through-holes is indicated in the left bottom corner for each image. The optical microscope images (do not correspond to the same SEM image) are presented next to SEM images.

We examined the top surface of the processed samples with a scanning electron microscope (SEM) Hitachi TM-1000 and an optical microscope Olympus BX51. We found that the craters appear somewhat different when changing the laser operation modes from kHz to GHz. SEM and optical microscope images of the ablated craters are presented in Figure 3. These through-holes were created with the 5 W average power laser setting on a 20 µm thick sample. The entrance-exit ratio of the holes is approximately 2:1 in all presented cases. The exits of the holes are not displayed since no significant differences (apart from variations in the diameter) from one mode to the other were evident. The greatest differences are visible at the top surface (entrance) of the holes. In addition, due to the chosen ratio criterion, the probability of material bulging or irregular crater appearance is minimized. As is evident, many laser shots were needed to produce the final hole after the threshold of piercing. At the threshold of the foil piercing the exit hole could have been irregular; however, as consecutive laser shots ablate the material, they also smoothen not only the walls of the hole but also the exit (bottom of the hole). The regular through-hole was evident in the profilometer measurements, and inspection with the high numerical aperture optical microscope showed regular holes on both entrance and exit surfaces. We should also mention that the beam profile exhibits good quality with an M^2^ criterion less than 1.1.

For the MHz case, a lot more sputtered material is visible in the vicinity of the crater, possibly caused by melt pool dislocation due to the shock waves generated after each pulse. Protrusions are formed with the high average power (5 W) setting in all cases and suggest that expulsion of the melted material has occurred after the set number of pulses was fired. Since all the burst pulses impinge the sample on the same location at a repetition rate of 100 kHz, the low-intensity peripheral regions of the beam heats the material without ablation, and consequently, heat accumulation occurs in those places. This effect is more pronounced for higher laser repetition rates [37]. Therefore we would suspect better quality through-holes produced with a laser whose repetition rate is lower and in the vicinity of 10–30 kHz. A protrusion of 5 µm is visible for the GHz case, whereas for the MHz case it is slightly smaller (<3 µm); however, sputtered micro-particles are visible around the crater over a circle with a radius of approx. 30–35 µm. The MACRO mode appears to generate the largest protrusion, which is estimated to be around 7 µm in length and has an uneven shape. Optical microscope images are shown in Figure 3 next to their corresponding SEM images. The images confirm that the GHz repetition rate case produces material discoloration that is more severe as compared to the results of the other repetition rate cases. Moreover, the area around the crater is colored, which is a typical case of oxide formation that cannot be removed by cleaning or washing. It is likely that the heat accumulation at the crater periphery is significant enough to induce oxide formation. The hole periphery appears darker when a 100 kHz repetition rate is used; however, this is typical ablation debris that can be cleaned off by mechanically wiping or washing in an ultrasonic bath. After cleaning, only the protrusion remains, thus confirming that the darkening is caused by small debris scattered around the impact area. Surprisingly, significantly less small-scale debris is visible for the MHz cases. We attribute it to generated multiple shock waves by individual pulses in the burst that clean the sample surface by throwing small debris further from the crater. The produced through-holes in the thicker samples are very similar to the ones presented in Figure 3, with proportionally (to their thickness) larger protrusions. It is worth remembering that all the experiments were conducted at the fundamental harmonic of 1030 nm. It is known that for Invar the absorption of light increases when the wavelength decreases. Higher absorption leads to a lower damage threshold as compared to the infrared case. For such wavelengths, it may be possible to drill through the material with a lower average power/pulse energy setting. In addition, as it was shown that micromachining throughput is higher for thicker materials (>50 µm) when the number of sub-pulses within the burst is lower, as compared to thinner materials (20 µm), for a shorter wavelength setting, it may be the case that it is more efficient to use a burst setting that splits a single pulse into an even higher number of sub-pulses. As the light penetration length is smaller for the shorter wavelength, it is expected that less thermal damage would be present. To this end, the micromachining of materials with different wavelengths remains a topic of future research.

## 4. Conclusions

We have presented results of metal (20–250 µm thick Invar (Fe64/Ni36) foils) percussion drilling experiments carried out with a multi-burst generating femtosecond laser system. Different burst modes were investigated that equate to GHz, MHz and kHz repetition rates. We have investigated the ablated volume per burst dependence on laser power. These results enable us to demonstrate that the efficiency of micromachining through-holes is increased when a certain repetition rate burst mode of a laser is used. We have found that the MHz repetition rate burst is almost an order of magnitude more efficient than the conventional laser case of 100 kHz for thin Invar samples (20 µm) and two times more efficient for thick samples (250 µm). The same investigation for the GHz repetition rate burst case shows that it is not as efficient as the MHz regime, though still approximately twice better (in terms of ablated volume per burst-pulse) than a typical 100 kHz femtosecond laser for thin samples, while the efficiency of ablation decreases for thick samples and is even lower than ablation with the 100 kHz laser. We attribute these differences to changing material properties when burst pulses impinge on the surface and apply theories proposed by multiple parties, namely that the decrease in fabrication efficiency for the GHz case is believed to be caused by the unwanted beam-plasma interaction effects (reignition, scattering). The efficiency decreases due to V-grove crater formation and lower fluence reaching the material surface. Overall, burst pulse generation and its use for micromachining shows a very promising direction for faster, more efficient micromachining while maintaining a similar micromachining quality.
